# PCR versus Hybridization for Detecting Virulence Genes of Enterohemorrhagic *Escherichia coli*

**DOI:** 10.3201/eid1308.060428

**Published:** 2007-08

**Authors:** Robert S. Gerrish, James E. Lee, June Reed, Joel Williams, Larry D. Farrell, Kathleen M. Spiegel, Peter P. Sheridan, Malcolm S. Shields

**Affiliations:** * Idaho State University, Pocatello, Idaho, USA

**Keywords:** Stx1, Stx2, eae, *ehl*x, HlyA, ToxB, ORF1, ORF2, KatP, EspP, bfp, EAF, pOSAK1, pB171, pO157, EHEC, virulence factors, dispatch

## Abstract

We compared PCR amplification of 9 enterohemorrhagic *Escherichia coli* virulence factors among 40 isolates (21 O/H antigenicity classes) with DNA hybridization. Both methods showed 100% of the chromosomal and phage genes: *eae*, *stx,* and *stx2*. PCR did not detect 4%–20% of hybridizable plasmid genes: *hly*A, *katP*, *espP*, *toxB*, open reading frame (ORF) 1, and ORF2.

Enterohemorrhagic *Escherichia coli* (EHEC) pathogenicity is usually linked to a Shiga toxin (*1*,*2*) and virulence factors, including adhesins, toxins, invasins, protein secretion systems, iron uptake systems, and several unidentified functions (*3*,*4*), which are unrelated to strain phylogeny. In many laboratories, sorbitol-MacConkey medium is commonly used to screen for the slow sorbitol fermentation phenotype of the most common Shiga toxin–containing strain: O157:H7 (*5*), but this process does not address the pathogenic potential of the remaining sorbitol-positive *E. coli*. These organisms can be detected by immunologic methods or PCR evaluation of virulence factors. PCR is the most useful method for virulence factor detection, and others have made convincing arguments for its use in characterizing the virulence factor patterns of potential pathogens (*6**,**7*).

Variation in virulence factor targets and use of different PCR primers contribute to variable results in detecting the most common virulence factors: *stx*1, *stx*2, *eae*, and *hlyA* (or *ehx*A). Variation in amplification success is likely to increase because more virulence factor variants are certain to emerge as more EHEC and Shiga toxin–producing *E. coli* (STEC) strains are identified. This study addresses the potential for a broad and well-characterized set of control strains relative to virulence factor amplification and confirmed by Southern hybridization.

## The Study

We used PCR amplification and Southern blot hybridization to detect 9 virulence factors among 40 EHEC type-strains from the STEC Center, National Food Safety and Toxicology Center, Michigan State University (East Lansing, MI, USA). The virulence factor targets were the following: 1 chromosomal (*eae* [*8*]), 2 phage (*stx*1 and 2), and 6 plasmid (open reading frame [ORF] 1, ORF2 of pOSAK1 [*1*,*2*]; espP [*9*], hlyA [*4*,*10*], katP [*11*], and tox B [*12*] of pO157) (Table). DNA-DNA hybridization probes were made from virulence factors amplified from O157:H7 EDL933 genomic DNA.

**Table:** Virulence factor targets and primers, including nucleotide sequences, reference, and PCR conditions*

Primer name	Nucleotide sequence (5′→3′)	Target (bp)	Ref.	PCR conditions		
				Denature	Anneal	Extension
STX1U	GTAACATCGCTCTTGCCACA	Stx1 gene (204)	This study	95°C, 60 s	53.7°C, 60 s	72°C, 240 s
STX1D	CGCTTTGCTGATTTTTCACA					
STX2U	GTTCCGGAATGCAAATCAGT	Stx2 gene (206)	This study	95°C, 60 s	53.7°C, 60 s	72°C, 240 s
STX2D	CGGCGTCATCGTATACACAG					
eae-1	ACGTTGCAGCATGGGTAACTC	Intimin (818)	(*8*)	95°C, 60 s	57.1°C, 60 s	72°C, 240 s
eae-2	GATCGGCAACAGTTTCACCTG					
ToxBF	TGGCCTTGCGCTCTATAAGAACCT	ToxB (823)	This study	95°C, 60 s	60°C, 60 s	72°C, 240 s
ToxBR	ACCACGCCGTGAGAATAATGTCCA					
HlyA1F	GGTGCAGCAGAAAAAGTTGTAG	HlyA (1551)	(*13*)	95°C, 60 s	55.5°C,60 s	72°C, 240 s
HlyA1R	TCTCGCCTGATAGTGTTTGGTA					
EspPF	CGGCAGAGTATCATCAAGAGC	EspP (397)	This study	95°C, 60 s	55.5°C, 60 s	72°C, 240 s
EspPR	CATTAAATGGAGTTATGCGTC					
KatPF	TTTAAAACGCTGGGATTTGC	KatP (1174)	This study	95°C,60 s	52.0°C, 60 s	72°C, 240 s
KatPR	CTCCTGAGAGGCGTCAGTTC					
MalBU	GACCTCGGTTTAGTTCACAGA	MalB promoter (414)	This study	95°C, 60 s	55.8°C, 60 s	72°C, 240 s
MalBDn	AGCGCGTAGGACTGAAACACCATA					
ORF1F	TTTTTCAAAGCAAATGATGTGG	ORF 1 pOSAK1 (385)	This study	95°C, 60 s	49.8°C, 60 s	72°C, 240 s
ORF1R	GGCGTAGCTAGGTTGAAATTATG					
ORF2F	CAA CCTAGCTACGCCACCAT	ORF 2 pOSAK1 (869)	This study	95°C, 60 s	54.3°C, 60 s	72°C, 240 s
ORF2R	CATCAGGCGGAAATACCACT					
EAF1	CAGGGTAAAAGAAAGATGATAA	Eaf (397)	(*14*)	95°C, 60 s	49.8°C, 60 s	72°C, 240 s
EAF2	TATGGGGACCATGTATTATCA					
BFP1	GATTGAATCTGCAATGGC	Bfp (597)	(*15*)	95°C, 60 s	51.6°C, 60 s	72°C, 240 s
BFP2	GGATTACTGTCCTCACATAT					

*Ref., reference; ORF, open reading frame.

PCR amplification was carried out with PCR primers (20 pmol/L each per 50 µL reaction) (Integrated DNA Technologies, Coralville, IA, USA) (Table) and 1 µL genomic DNA (extracted from overnight Luria-Bertani broth cultures according to PureGene DNA isolation kit instructions [Gentra Systems, Minneapolis, MN, USA] and dissolved in 50 µL 10 mmol/L Tris, pH 8.3) in a PCR cocktail containing 1× PCR buffer, 1.5 mmol/L MgCl_2_, 1 U Vent exo(–) polymerase from New England BioLabs (Beverly, MA, USA), and 200 µmol/L each dATP, dGTP, dTTP, and dCTP. The mix was incubated for 30 cycles of 94°C, 40 s; annealing (for temperatures, see Table), 45 s; 72°C, 60 s, and a final 10-min extension at 72°C. Amplification products were confirmed by DNA sequencing.

^32^P-labeled DNA probes were made from 2 µg PCR amplicons (purified by Montage PCR Cleanup Spin Column (Milipore Corp., Burlington, MA, USA). The DNA was denatured at 94°C, 40 sec; annealed (temperatures in Table) with 50 pmol/L of the appropriate PCR primers, 45 s extended for 2 h at 72°C. The 1× buffer contained the following: 1.5 mmol/L MgCl_2;_ 0.4 mmol/L each dATP, dGTP, dTTP; 2.0 µL 3,000 Ci/mmol α-^32^P-dCTP (MP Bioscience, Buxton, UK); and 1.25 U Taq polymerase in a 50 µL final volume. Unincorporated ^32^P-nucleotide was removed by Sephadex G-50 in Tris-EDTA, 1% sodium dodecyl sulfate (SDS).

Bacteria (800 µL overnight cultures) were transferred to Hybond-N+ nitrocellulose membrane (Amersham Biosciences UK Ltd, Buckinghamshire, UK) by dot-blot vacuum filtration apparatus (Schleicher and Schuell, Keene, NH, USA). Lysis and binding of genomic DNA fixation were carried out by exposure to lysis solution (1.5 mol/L NaCl, 0.5 mol/L NaOH) twice for 5 min each, and twice with neutralization solution (1 mol/L Tris-Cl, pH 7.4; 1.5 mol/L NaCl) for 5 min each. The filter was then submerged in 2× SSC with gentle agitation, air dried, and the DNA UV (254 nm) cross-linked at 120,000J/cm^2^ (CL-1000 cross-linker, Fisher Biotech, Pittsburgh, PA, USA).

Probe hybridization was carried out in rotating hybridization bottles (Fisher Scientific Isotemp hybridization oven, Fisher Biotech) in 20 mL 6× SSC, 1% SDS at 68°C. Membranes were washed twice, for 1 min, in room temperature 2× SSC, 0.1% SDS, and twice at 45°C for 1 h in 1× SSC, 1% SDS. Hybridized membranes were exposed overnight with a phospho-imaging screen (Bio-Rad, Hercules, CA, USA) and visualized with a Personal Molecular Imager FX (Bio-Rad). The 3 chromosomal targets (*stx*1, *stx*2, and *eae*) were detected with 100% efficiency by both PCR and hybridization (no. positive by PCR/no. positive by hybridization): 21/21 *stx*I, 19/19 *stx*II, and 37/37 *eae*. Plasmid-associated genes, however, were detected with less efficiency relative to hybridization: *katP*: 15/17 (88%), *hly*A: 26/27 (96%), *espP*: 19/23 (83%), *toxB*: 13/16 (81%), and both ORF1 and ORF2: 4/5 (80%) (Appendix Table).

Seventy-five percent (30/40) of the pathogenic *E. coli* strains tested contained at least 1 *stx* gene, 23% (9/40) were positive for both *stx*1 and *stx*2. The most common gene detected was intimin (*eae*), which was positive by both PCR and hybridization in 37/40 (93%) of the strains. While *eae* is strongly correlated with Shiga toxin, the adherence phenotype conveyed may be sufficient to cause a pathogenic state because 4 of the clinical isolates investigated contained only the *eae* gene.

Six plasmid virulence factor genes of pO157 and pOSAK1were targeted. Thirty-one (78%) of the 40 pathogenic strains tested were positive for at least 1 (by hybridization, PCR, or both) of the 4 genes, *toxB*, *katP, hlyA*, *espP*, which are usually carried on the archetypal pO157 plasmid: 11/31 (35%) retained all 4, 4/31(13%) carried three, 9/31 (29%) carried 2, and 5/31 (16%) carried only 1 (the sole PCR-positive/hybridization-negative isolate [*esp*P in ED-31] was presumed to result from a nonspecific amplification).

Five EHEC strains (13%) hybridized to both ORF1 and ORF2 (from pOSAK1) (*1*,*4*), but only 4 were amplifiable. These same 4 also contained *eae* and at least 1 Shiga toxin gene (the 1 that failed to amplify [E851/71] lacked both *stx*1 and *stx*2).

## Conclusions

The current accepted standard for EHEC identification is amplification of *stx1*, *stx2*, *eae*, and *hlyA* by PCR. However, this technology is generally only available at large hospital or state health laboratories. Hybridization is superior to culture screening methods and largely complimentary to PCR, but has a potentially broader epidemiologic application since it is unaffected by minor sequence variations that can completely inhibit PCR.

Only 3% of the 360 virulence factor hybridizations made in this study did not amplify. PCR failure is expected with its relatively higher sensitivity to single base primer-hybrid mismatch compared to whole amplicon hybridization. Notably, however, all 12 variations detected were among plasmid-associated virulence factors: 95% (228/240) of the plasmid hybridizable targets were amplified, compared to 100% (120/120) of the hybridizable chromosomal targets.

Although we detected the variable presence of genes ostensibly associated with 2 plasmids (pO157 and pOSAK1) and the bacterial chromosome, we did not attempt to verify either plasmid or chromosomal locations for any of the amplicons or DNA:DNA hybrids. While all virulence factor targets summarized in this study are subject to change there have been reports of any of the putative chromosomal or plasmid virulence factor targets in this study being found elsewhere.

Prager et al. (*7*) recently reported, using PCR alone, a wide variety of 25 virulence factor combinations among 266 pathogenic *E. coli* isolates representing 81 serotypes. Such diversity speaks directly to the need to accurately assess virulence factor presence to evaluate epidemiologic and clinical correlations. A similar 5% failure of the plasmid-associated virulence factor amplifications could have implications in such virulence factor correlations. Overall, however, these results are very similar to those of this study of prospective control strains. The use of a single control, such as EDL 933, will inherently bias PCR detection schemes since a failure of amplification in a test will be read as the absence of virulence factor element because it was amplifiable in the control.

If amplification failure is a measure of template variation, we find a much greater variability among plasmid-associated virulence factors. Although pO157 has been reported in most O157 H7 strains (*13*), our study demonstrates a high variability in the putative virulence factor content of pO157 as well as a highly variable content of pO157-associated virulence factors among the O157 isolates screened. Finally, pO157-associated virulence factors were detected among all but 4 of the 20 *E. coli* serotypes examined.

## Supplementary Material

Appendix Table Hybridization/PCR amplification results for 9 virulence genes*dagger

## References

[1] Nataro JP, Kaper JB. Diarrheagenic *Escherichia coli.* Clin Microbiol Rev. 1998;11:142–201. 9457432 10.1128/cmr.11.1.142 PMC121379

[2] Makino S, Tobe T, Asakura H, Watarai M, Ikeda T, Takeshi K, Distribution of the secondary type III secretion system locus found in enterohemorrhagic *Escherichia coli* O157:H7 isolates among Shiga toxin–producing *E. coli* strains. J Clin Microbiol. 2003;41:2341–7. 10.1128/JCM.41.6.2341-2347.2003 12791847 PMC156528

[3] Hacker J, Kaper JB. Pathogenicity islands and the evolution of microbes. Annu Rev Microbiol. 2000;54:641–79. 10.1146/annurev.micro.54.1.641 11018140

[4] Burland V, Shao Y, Perna NT, Plunkett G. Sofia HJBlattner FR. The complete DNA sequence and analysis of the large virulence plasmid of *Escherichia coli* O157:H7. Nucleic Acids Res. 1998;26:4196–204. 10.1093/nar/26.18.4196 9722640 PMC147824

[5] Farmer JJ III, Davis BR. H7 antiserum-sorbitol fermentation medium: a single tube screening medium for detecting *Escherichia coli* O157:H7 associated with hemorrhagic colitis. J Clin Microbiol. 1985;22:620–5. 3908474 10.1128/jcm.22.4.620-625.1985 PMC268479

[6] Osek J. Development of a multiplex PCR approach for the identification of Shiga toxin–producing *Escherichia coli* strains and their major virulence factor genes. J Appl Microbiol. 2003;95:1217–25. 10.1046/j.1365-2672.2003.02091.x 14632994

[7] Prager R, Annemuller S, Tschape H. Diversity of virulence patterns among Shiga toxin–producing *Escherichia coli* from human clinical cases-need for more detailed diagnostics. Int J Med Microbiol. 2005;295:29–38. 10.1016/j.ijmm.2004.12.009 15861814

[8] Blanco JE, Blanco M, Blanco J, Mora A, Balaguer L, Mourino M, O serogroups, biotypes, and *eae* genes in *Escherichia coli* strains isolated from diarrheic and healthy rabbits. J Clin Microbiol. 1996;34:3101–7. 8940455 10.1128/jcm.34.12.3101-3107.1996 PMC229466

[9] Brunder W, Schmidt H, Frosch M, Karch H. The large plasmids of Shiga-toxin–producing *Escherichia coli* (STEC) are highly variable genetic elements. Microbiology. 1999;145:1005–14. 10.1099/13500872-145-5-1005 10376815

[10] Makino K, Ishii K, Yasunaga T, Hattori M, Yokoyama K, Yutsudo CH, Complete nucleotide sequences of 93-kb and 3.3-kb plasmids of an enterohemorrhagic *Escherichia coli* O157:H7 derived from Sakai outbreak. DNA Res. 1998;5:1–9. 10.1093/dnares/5.1.1 9628576

[11] Brunder W. Schmidt H, Karch H. KatP, a novel catalase-peroxidase encoded by the large plasmid of enterohaemorrhagic *Escherichia coli* O157:H7. Microbiology. 1996;142:3305–15. 10.1099/13500872-142-11-3305 8969527

[12] Tatsuno I, Horie M, Abe H, Miki T, Makino K, Shinagawa H, toxB gene on pO157 of enterohemorrhagic *Escherichia coli* O157:H7 is required for full epithelial cell adherence phenotype. Infect Immun. 2001;69:6660–9. 10.1128/IAI.69.11.6660-6669.2001 11598035 PMC100040

[13] Schmidt H, Beutin L, Karch H. Molecular analysis of the plasmid-encoded hemolysin of *Escherichia coli* O157:H7 strain EDL 933. Infect Immun. 1995;63:1055–61. 7868227 10.1128/iai.63.3.1055-1061.1995 PMC173109

[14] Franke J, Franke S, Schmidt H, Schwarzkopf A, Wieler LH, Baljer G, Nucleotide sequence analysis of enteropathogenic *Escherichia coli* (EPEC) adherence factor probe and development of PCR for rapid detection of EPEC harboring virulence plasmids. J Clin Microbiol. 1994;32:2460–3. 7814482 10.1128/jcm.32.10.2460-2463.1994 PMC264083

[15] Wieler LH, Vieler E, Erpenstein C, Schlapp T, Steinruck H, Bauerfeind R, Shiga toxin-producing *Escherichia coli* strains from bovines: association of adhesion with carriage of *eae* and other genes. J Clin Microbiol. 1996;34:2980–4. 8940434 10.1128/jcm.34.12.2980-2984.1996 PMC229445

