# Supplementary material for: PCR versus Hybridization for Detecting Virulence Genes of Enterohemorrhagic Escherichia coli
Source: Emerg Infect Dis. 2007 Aug;13(8):1253–5. doi: 10.3201/eid1308.060428 (PMC2828064; doi:10.3201/eid1308.060428)
Supplement: Appendix Table — Hybridization/PCR amplification results for 9 virulence genes*dagger [file 06-0428_appT-s1.pdf]

Appendix Table. Hybridization/PCR amplification results for 9 virulence genes*†

	Antigen			Chromosomal genes			pO157 genes				pOSAK1 genes	
Escherichia coli strain	O	H	Sorbitol fermentation‡	*Stx1*	*Stx2*	*eae*	*KatP*	*ToxB*	*HlyA*	*EspP*	*ORF1*	*ORF2*
K12§				-/-	-/-	-/-	-/-	-/-	-/-	-/-	-/-	-/-
EDL933	157	7	–	+/+	+/+	+/+	+/+	+/+	+/+	+/+	+/+	+/+
E851/71	142	6		-/-	-/-	+/+	-/-	+/-	-/-	-/-	+/-	+/-
C771	142	6		-/-	-/-	+/+	+/-	+/-	-/-	-/-	-/-	-/-
93–111	157	7	–	+/+	+/+	+/+	+/+	+/+	+/+	+/+	-/-	-/-
OK–1	157	7	–	+/+	+/+	+/+	+/+	+/+	+/+	+/+	+/+	+/+
86–24	157	7	–	-/-	+/+	+/+	+/+	+/+	+/+	+/+	+/+	+/+
2886–75	157	7	–	+/+	-/-	+/+	+/-	+/+	+/+	+/+	+/+	+/+
493 /89	157	–	+	-/-	+/+	+/+	-/-	-/-	+/+	-/-	-/-	-/-
E32511	157	–	–	-/-	+/+	+/+	+/+	+/+	+/+	+/+	-/-	-/-
G5101	157	7	–	+/+	+/+	+/+	-/-	+/+	+/+	-/-	-/-	-/-
5905	55	7	+	-/-	+/+	+/+	-/-	-/-	-/-	-/-	-/-	-/-
TB182A	55	7	–	-/-	-/-	+/+	+/+	-/-	-/-	-/-	-/-	-/-
DEC5D	55	7	+	-/-	-/-	+/+	-/-	-/-	-/-	-/-	-/-	-/-
3256–97	55	7	+	-/-	+/+	+/+	-/-	-/-	-/-	-/-	-/-	-/-
DEC8B												
111	8	+	+/+	+/+	+/+	+/+	-/-	+/+	+/-	-/-	-/-	
3007–85	111	–	+	+/+	+/+	+/+	-/-	-/-	+/+	+/-	-/-	-/-
TB226A	111	–	+	-/-	+/+	+/+	+/+	-/-	+/+	+/-	-/-	-/-
928/91	111	–	+	+/+	+/+	+/+	-/-	-/-	-/-	-/-	-/-	-/-
412/55	111	–	+	+/+	-/-	+/+	-/-	-/-	-/-	-/-	-/-	-/-
DEC8C	111	–		+/+	-/-	+/+	+/+	+/+	+/+	+/+	-/-	-/-
C412	111	–	+	+/+	-/-	+/+	+/+	-/-	+/+	+/-	-/-	-/-
H19	26	11	+	+/+	-/-	+/+	+/+	+/+	+/+	+/+	-/-	-/-
DEC10B	26	11	+	+/+	-/-	+/+	-/-	-/-	+/-	-/-	-/-	-/-
DEC10C	26	11	+	-/-	-/-	+/+	+/+	+/+	+/+	+/+	-/-	-/-
TB285C	26	–	+	+/+	-/-	+/+	+/+	+/+	+/+	+/+	-/-	-/-
VP30	26	–	+	-/-	-/-	+/+	+/+	+/+	+/+	+/+	-/-	-/-
RDEC–1	15	–	+	-/-	-/-	+/+	-/-	-/-	-/-	-/-	-/-	-/-
BCL19	N	–	+	+/+	-/-	+/+	-/-	-/-	+/+	+/+	-/-	-/-
DEC10J	70	11		+/+	-/-	+/+	-/-	+/+	+/+	+/+	-/-	-/-
ED–31	111	–	+	+/+	+/+	+/+	-/-	-/-	-/-	–/+	-/-	-/-
CL–3	113	21	+	-/-	+/+	-/-	-/-	-/-	+/+	+/+	-/-	-/-
DEC16A	113	21	+	-/-	-/-	+/+	-/-	-/-	-/-	-/-	-/-	-/-
G5506	104	21	–	-/-	+/+	+/+	-/-	-/-	+/+	+/+	-/-	-/-
B2F1	91	21	–	-/-	+/+	+/+	-/-	-/-	+/+	+/+	-/-	-/-
23/67	91	7	+	-/-	-/-	+/+	-/-	-/-	-/-	-/-	-/-	-/-
TB154A	103	6	+	+/+	-/-	+/+	-/-	-/-	+/+	-/-	-/-	-/-

88–1509	15	27	+	+/+	+/+	-/-	-/-	-/-	-/-	-/-	-/-	-/-
M2113	156	21	+	+/+	-/-	+/+	-/-	-/-	+/+	+/+	-/-	-/-
BCL17	5	–	+	+/+	-/-	+/+	+/+	+/-	+/+	+/+	-/-	-/-
90–1787	X03	–	+	-/-	+/+	-/-	-/-	-/-	+/+	+/+	-/-	-/-
Total positive (Hyb+/PCR)				21/21	19/19	37/37	17/15	16/13	27/26	23/20	5/4	5/4

*Forty enterohemorrhagic *Escherichia coli* isolates representing 21 distinct OH antigenicity groups obtained from the National Food Safety and Toxicology Center, Michigan State University.

†ORF, open reading frame; hyb, hybridized.

‡Fermentation of sorbitol as determined by zorbitol–MaConkey plates.

§*E. coli* strain DH5a.
